# Resident-to-resident aggression in Norwegian nursing homes: a cross-sectional exploratory study

**DOI:** 10.1186/s12877-020-01623-7

**Published:** 2020-06-24

**Authors:** Anja Botngård, Arne Henning Eide, Laura Mosqueda, Wenche Malmedal

**Affiliations:** 1 Department of Public Health and Nursing, Norwegian University of Science and Technology, Trondheim, Norway; 2 Department of Health Research, SINTEF Digital, Oslo, Norway; 3 Department of Family Medicine, Keck School of Medicine of the University of Southern California, Los Angeles, USA

**Keywords:** Resident-to-resident aggression, Nursing homes, Long-term care settings, Nursing care

## Abstract

**Background:**

Resident-to-resident aggression in nursing homes is a public health problem of growing concern, impacting the safety, health and well-being of all residents involved. Despite this, little research has been conducted on its occurrence particularly in large-scale national studies. The aim of this study was to explore the extent and nature of resident-to-resident aggression in Norwegian nursing homes, as reported by nursing staff.

**Methods:**

We conducted a cross-sectional exploratory study, where nursing staff in 100 randomly selected Norwegian nursing homes completed a pen and paper survey measuring how often they had observed incidents of resident-to-resident aggression during the past year. These rates were separated according to nursing home size, location and units of workplace.

**Results:**

Of the 3693 nursing staff who participated (response rate 60.1%), 88.8% had observed one or more incidents of resident-to-resident aggression during the past year, with acts of verbal and physical aggression being the most commonly reported. Nursing staff working in dementia special care units, larger nursing homes and nursing homes located in suburban/urban municipalities, reported more incidents of resident-to-resident aggression than staff in short-term and long-term units, small institutions, and nursing homes located in rural municipalities.

**Conclusions:**

This is the first national study of resident-to-resident aggression in Norwegian nursing homes and is one of the largest surveys worldwide exploring the extent and nature of resident-to-resident aggression in long-term care settings. Overall, we found a high occurrence of all types of aggression, suggesting a need for strategies to improve residents’ safety and quality of life in nursing homes.

## Background

Aggression between residents in long-term care settings is a public health problem of growing concern, impacting the safety, health and well-being of all residents involved [1, 2]. Compared to research on elder abuse committed by healthcare staff [3], and the violence directed toward caregivers by nursing home residents [4–7], few studies have examined the occurrence of aggression that occurs *between* residents in long-term care facilities [2, 8, 9]. Such aggression has been associated with a range of serious health consequences, from minor bruises to fatal injuries, psychological distress, poorer quality of life, and an increased risk of hospitalisations and premature death [2, 8, 10–12]. Resident-to-resident aggression may also create an unsafe and stressful working environment for healthcare staff [8, 13].

The Centers for Disease Control and Prevention (CDC) do not define aggression between nursing homes residents as a form of elder abuse [14], and *“the term “abuse” implies intent on the part of the initiator, which might not be the case in situations where the perpetrator lacks capacity (*e.g.*, as seen with dementia residents)”* [1]. The term “abuse” may also be more stigmatising than “aggression” and hence contribute to concerns of underreporting [1]. Previous research has used different terms to describe residents displaying aggressive behaviours such as “exhibitors” [11, 15], “perpetrators” [1, 16], “initiators” [2, 17], and “aggressors” [10]. Furthermore, prior research has used a variety of terminologies including resident-to-resident abuse [2, 18–20], resident-to-resident (elder) mistreatment [9, 16, 21–26], resident-to-resident relational aggression [27], resident-to-resident violence [10, 17, 28], and resident-to-resident (physical) aggression [8, 12, 13, 15, 29–32]. In 2015, a consensus-building workshop with an expert panel of researchers and practitioners reached an agreement on the term resident-to-resident aggression (RRA) defined as: *“negative, aggressive and intrusive verbal, physical, sexual, and material interactions between long-term care residents that in a community setting would likely be unwelcome and potentially cause physical or psychological distress or harm to the recipient”* [1].

One of the first studies on RRA was conducted in 2004 by Shinoda-Tagawa and colleagues, who performed a case-control study of the Minimum Data Set assessments for nursing home residents and of the Massachusetts Department of Public Health’s Complaint and Incident Reporting System, to assess risk factors of resident injury inflicted by co-residents [10]. Since then, researchers have used different study approaches to examine the extent, nature and associations including secondary analysis of existing records/registers [12, 15, 16, 31, 33] and publicly available data (media) [11], qualitative event reconstructions [30], observational designs [9], and interviews or surveys of staff [13, 17, 18, 27, 34, 35], family members [19, 20], and/or residents themselves [13, 27].

A study by Lachs et al. [9] estimated the prevalence of RRA based on resident and staff interviews, shift coupons, event logs, incident/accident reports and forensic chart interviews, and found that 20.2% of residents had been involved in at least one incident or RRA during a one-month period [9]. The same study reported that 46.9% had screamed at and 11.3% had hit co-residents [9]. Another study by Castle et al. [18] found that 97% of nursing staff had observed residents yelling and cursing, and 94% of staff had observed residents pushing, grabbing or pinching co-residents during a three-month period.

To prevent and manage resident-to-resident aggression, it is important to understand contributing risk factors and situational triggers; several researchers have used a social-ecological approach, emphasising that RRA is shaped by individual characteristics (victim and aggressor) as well as the physical and social environment in which they live [20, 30]. Pillemer et al. [30] highlighted that “*the needs, person-environment fit, and antecedents or consequences for both members of the RRA dyad must all be considered in order to better understand the influences that contribute to aggressive behaviour”.*

Previous research has found that victims of RRA are both males [10] and females [16, 19], cognitively impaired [10, 13, 16], and/or they often demonstrate neuropsychiatric symptoms (NPS) such as agitation, aggression and/or wandering (getting in harm’s way) [10, 13]. Aggressors are more likely to be male [11, 12, 15, 31], younger than their victims [11, 12, 15], intolerant of residents with cognitive impairments [16], more physically dependent [10], and/or they suffer from cognitive impairment, dementia or mental illness themselves [10–12]. A higher incidence of RRA has been found in larger compared to smaller nursing homes [12], institutions located in metropolitan rather than in non-metropolitan areas [12], and in dementia special care units compared to other units [10]. Some studies have reported that RRA is most often exhibited in shared dining/living rooms or hallways [12, 13, 15, 16], while others have found incidents to be more prevalent in residents’ rooms [10, 11, 13, 16]. Most episodes occur in the afternoon or evening [12, 13, 16], and often when staff members are not present [11, 15, 36].

The completion of this national study will provide new knowledge on the magnitude of resident-to-resident aggression, so appropriate strategies to prevent and manage RRA can be established and evaluated. The objectives of the present study were to 1) examine the extent and nature of resident-to-resident aggression in Norwegian nursing homes and 2) explore differences in facility characteristics between nursing homes with a high and low occurrence of RRA.

## Methods

### Study design

This study was a cross-sectional exploratory survey of nursing staff in Norwegian nursing homes, carried out between October 2018 and January 2019. The survey was part of a larger national study where the aim was to measure the occurrence of different types of abuse/aggression in nursing homes; staff-to-resident abuse, relative-to-resident abuse and resident-to-resident aggression. In this article we will present findings on resident-to-resident aggression. The prevalence of staff-to-resident abuse is reported elsewhere [37].

### Setting

In Norway, municipalities own and operate the vast majority of nursing homes, defined as a health institution that provides patients with 24-h stay, treatment and care that do not need to be conducted in hospitals, but which still require more care than is possible to provide in the patient’s own home [38]. Norwegian nursing homes contain both short- and long-term units and are mostly managed by registered nurses (RNs) in collaboration with a physician [39]. Seventy-four percent of residents in long-term units are 80 years or older, 80% have cognitive impairments, and four out of five residents require extensive need for assistance [40, 41]. An increasing number of municipalities have established special care units specifically designed for people with dementia with severe neuropsychiatric symptoms [42]. These units are licensed in the same way as the other nursing home units, but often comprise fewer beds and a higher staff/resident-ratio [42].

### Sample size and randomisation

We were unable to statistically compute a sample size, because there exist few large surveys of resident-to-resident aggression and staff-to-resident abuse. However, in Ireland, they conducted a national survey on staff-resident interactions and conflicts which included 64 (out of 613) nursing homes and distributed 3000 questionnaires [43]. We therefore targeted a sample of about 10% (*n* = 100) of all nursing homes in Norway (*n* = 939). We used a computerised random number generator to select the sample of institutions registered as private or public nursing homes/retirement homes (hereafter called nursing homes or NH) in the Central Register of Establishments and Enterprises. We also randomly selected 50 institutions who could serve as reserves.

### Participants

Eligible participants were nursing staff who provided direct patient care during the three-week period of data collection. We included staff working full- or part-time on all shifts 24-h a day.

Of nursing staff in Norwegian nursing homes, 31% are registered nurses, 2.5% social educators/disability nurses, 42.5% licensed practical nurses, and 24% nursing assistants [41]. Registered nurses and social educators/disability nurses complete a three-year bachelor’s degree. Licensed practical nurses undergo a two-year high school programme with mentored training and practice [39]. Education about dementia care are provided in these programmes. Nursing assistants has no formal health education and are only trained by their nursing home employers [44].

### Recruitment

We recruited institutions by emailing invitations to each nursing home director, which was followed by a telephone call from the main author. Participation was voluntary and directors who agreed to participate sent a written consent by email with the potential number of participants and the name of one “coordinator” who could administer the study on site. This task was either assigned to ward managers, the nursing home directors or other staff appointed by directors. Of the 100 initially invited institutions, 27 declined to participate. In Norway, a median size nursing homes has 34 beds; in our initial recruitment phase, a disproportionate number of nursing homes with more than 34 beds rejected participation. To prevent further skewedness, we therefore invited the 30 largest nursing homes from the reserve list. A total of 6337 nursing staff were eligible for inclusion and 3811 returned questionnaires, giving a response rate of 60.1%. Of these, 118 staff members were excluded because they did not work in direct care, worked in day care centres or assisted living facilities, or had not answered any items concerning aggression/abuse. The remaining 3693 participants were included in the analysis, giving an analytic response rate of 58.3%. The flowchart of randomisation and recruitment is shown in Botngård et al. [37].

### Study variables and measurements

The primary outcome measure was the extent and nature of all forms of resident-to-resident aggression during the past year; verbal (i.e. criticising, humiliating, threatening), physical (i.e. pushing, kicking, hitting), material (i.e. stealing money/possessions, destroying property) and sexual (i.e. unwelcome touching, discussion of sexual activity, penetration). Estimates of aggression were separated according to nursing home size, location and units. Nursing homes with 50 or fewer beds were considered small, and institutions with more than 50 beds were considered large; the same cut-off value has been used in other studies [43, 45]. The location of municipalities in which the participating nursing homes were situated was specified according to Statistics Norway’s centrality measures of municipalities. This is an index reflecting the degree of centrality based on inhabitants’ travel time to workplaces and service functions, where level one covers the most central municipalities (biggest cities) and level six the least central (rural villages) [46]. We categorized these levels into three groups: urban (level 1–2), suburban (level 3–4), and rural (level 5–6). Nursing home units in which the participants worked were short- and long-term and dementia special care units.

#### Measuring resident-to-resident aggression

We translated, modified and used a survey questionnaire developed in the United States (US) by Dr. Nicholas Castle, with his permission. This questionnaire has previously been used in four large surveys of staff to measure staff-to-resident abuse and resident-to-resident aggression in nursing homes and assisted living facilities [18, 35, 47, 48]. However, this questionnaire has not been validated in the context of resident-to-resident aggression. To the best of our knowledge, no other instruments exist that have measured both staff-to-resident abuse and resident-to-resident aggression in the same study, which was the purpose of this national survey of Norwegian nursing home staff. The description of the original questionnaire [35], translation process and modification of survey instrument, and the pilot study is described in our article of staff-to-resident abuse [37]. The final survey questionnaire contained 23 items measuring how often staff had observed residents committing acts of verbal aggression (7 items), physical aggression (7 items), material aggression (4 items), and sexual aggression (5 items) towards co-residents during the past year, with the following ordinal scale: “Never”, “Once”, “2–5 times”, “6–10 times”, and “More than 10 times”. Similar scoring values have been used to measure the annual prevalence of staff-to-resident abuse [43, 49, 50] and family violence [51]. The Cronbach’s alpha coefficients were 0.9 for verbal aggression and 0.9 for physical aggression. For financial/material and sexual aggression, the alpha coefficients were 0.5, which may be caused by our skewed results (towards “Never”). Nursing home directors completed one short questionnaire concerning facility characteristics.

### Data collection

Each nursing home was provided with instruction letters, survey questionnaires with an invitation letter on the first page, and sealed collection boxes. The instruction letter described in detail how the coordinators should administer the survey on site, and the first author contacted all coordinators by phone during the data collection period. No incentive was given directly to participants, but we offered an economic incentive to the eight institutions that achieved the highest response rate, where approximately 900 GBP was dedicated to staff welfare.

### Ethical considerations

Participation in the survey was voluntary and nursing home directors who agreed to participate sent a written consent by email to the first author. Participating nursing staff did not write their name or birth date on the questionnaire, and consent from staff was implied upon completion of the survey; when they placed the questionnaire in the sealed collection boxes. They were informed that they could not withdraw their participation after the questionnaire was returned. Each nursing home was assigned a unique code for data analyses. Participants were guaranteed that this code was kept safe by the first author only, and that no one could be identified in any publications. We applied the Regional Ethic Committee for Medical Research, and they approved the study in May 2018, reference number: 2018/314.

### Statistical analysis

Data were analysed with Stata 16.1 software package. Descriptive statistics of nursing staff and nursing homes are presented with percentages, means and standard deviations (SD). The Shapiro-Francia test was used to examine the normality of the dependent variable “Aggression”, where none of the items were found to be normally distributed (*p* < 0.05). Many items were skewed towards “Never”, so we dichotomised the dependent variable to “No aggression” (never) and “Aggression” (one or more incidents). All items under each subtype of aggression are summarised and presented in the text as percentages expressing the number of staff answering positive (“Aggression”) on at least one item. Pearson’s Chi-square test was conducted to examine the association between facility characteristics and the occurrence of all types of aggression.

Verbal and physical aggression provided some level of distribution, so we created a “chronicity” scale; number of times the set of acts in the scale occurred, among those who had observed one or more acts [51, 52]. This operationalisation of chronicity is often used to deal with skewed distributions when measuring violence [51, 53]. To create this scale, we added midpoints for the response categories as follows: “Once” = 1; “2–5 times” = 3.5; “6–10 times” = 8; “More than 10 times” = 12.5, before all items under each subtype were summed and presented with median and range (Table 2) [51]. A Kruskal-Wallis test was conducted to examine this difference in chronicity score (median) of verbal and physical aggression according to facility characteristics. Missing variables were removed. Considering the large sample size, we did not add any design- or post-stratification weights.

## Results

### Participant characteristics

Of the participating nursing staff, 91.5% were women, with a mean age of 41.3 years (SD 14.0), 42.6% were licensed practical nurses (high school education), 53.9% worked part-time, and 63.7% worked in long-term care units (Table 1). The nursing homes ranged in size from eight to 161 beds, where 63% were considered small with 50 beds or less. Forty-two percent of nursing homes were in suburban municipalities, and 94% were publicly owned and run by the municipalities.

**Table 1:** Characteristics of nursing staff and nursing homes

Characteristics	n (%)	Mean (SD)
**NURSING STAFF (*N = 3693*)**		
**Gender**		
Female	3362 (91.5)	
Male	312 (8.5)	
**Age (years)**		41.3 (14.0)
**Professional occupation**		
Assistant (no formal health education)	1023 (28.1)	
Licensed practical nurse	1553 (42.6)	
Registered nurse/social educator	1070 (29.3)	
**Working time**		
Full-time (≥35 h per week)	1503 (46.1)	
Part-time (< 35 h per week)	1757 (53.9)	
**Unit of workplace**		
Long-term care units	2243 (63.7)	
Dementia special care units	766 (21.8)	
Short-term care units	511 (14.5)	
**NURSING HOMES (*N = 100*)**		
**Facility size (number of beds)**		
Small (≤50 beds)	63 (63.0)	
Large (< 50 beds)	37 (37.0)	
**Location of municipalities**		
Urban (level 1–2)	31 (31.0)	
Suburban (level 3–4)	42 (42.0)	
Rural (level 5–6)	27 (27.0)	
**Ownership**		
Public	94 (94.0)	
Private	6 (6.0)	

### The extent and nature of resident-to-resident aggression

The total proportion of nursing staff who had observed at least one incident of resident-to-resident aggression during the past year was 88.8% (3010/3389). Among the different subtypes, 88.0% (3082/3501) of staff had observed verbal aggression, 69.4% (2473/3565) had observed physical aggression, 24.8% (896/3612) had observed material aggression, and 18.6% (672/3605) had observed sexual aggression at least once during the past year.

Table 2 illustrates nursing staff observations of resident-to-resident aggression during the past year. The most frequently reported acts of verbal aggression were residents arguing (79.1%), yelling (74.7%), and making nasty remarks (69.0%). Regarding physical aggression, the most commonly reported acts were residents behaving aggressively towards other residents (57.4%), bullying (46.8%) and pushing, grabbing or pinching (46.1%). The most prevalent acts of material aggression were residents stealing things (21.3%) and destroying other residents’ things (10.1%), while the most prevalent acts of sexual aggression were unwelcome touching (13.5%) and unwelcome remarks of sexual activity (11.5%). Furthermore, 0.61% of staff had observed incidents of digital penetration (e.g. finger) and 0.25% of staff had observed rape.

**Table 2:** Frequency and chronicity score of resident-to-resident aggression (*N* = 3693)

Type of aggression:		How often observed the past year (%):					
		N	Never	Once	2–5 times	6–10 times	> 10 times
**Verbal**	Yelling	3650	25.3	8.6	23.8	13.4	28.9
	Nasty remarks	3636	31.0	9.5	24.9	11.9	22.7
	Swearing	3650	46.9	8.5	19.3	9.5	15.8
	Humiliating remarks	3606	42.2	10.9	22.8	9.0	15.1
	Arguing	3648	20.9	9.5	27.2	12.6	29.8
	Threatening remarks	3630	59.2	9.1	14.2	6.3	11.2
	Critical remarks	3637	36.5	11.2	24.1	9.4	18.8
**Physical**	Pushing, grabbing, or pinching	3633	53.9	12.3	18.0	7.9	7.9
	Pulling hair or kicking	3629	77.4	7.0	9.0	3.2	3.4
	Purposely hurting	3635	82.9	6.1	7.0	2.0	2.0
	Throwing things at a resident	3633	75.0	10.2	9.5	2.5	2.8
	Hitting	3630	66.2	11.1	13.9	4.3	4.5
	Bullying	3636	53.2	9.9	18.7	7.4	10.8
	Behaving aggressively towards a resident	3636	42.6	11.9	23.6	9.0	12.9
**Material**	Stealing money	3636	98.0	1.1	0.6	0.2	0.1
	Stealing things	3637	78.7	5.0	9.0	3.2	4.1
	Signing documents without permission	3631	99.9	0.07	0.03	–	–
	Destroying a resident’s things	3640	89.9	3.3	4.4	1.2	1.2
**Sexual**	Unwelcome touching	3637	86.5	4.5	6.0	1.5	1.5
	Unwelcome discussion of sexual activity	3636	88.5	3.7	5.2	1.4	1.2
	Exposure of a resident’s private-body parts	3627	98.7	0.6	0.3	0.2	0.2
	Digital penetration (e.g. finger)	3632	99.39	0.36	0.17	0.08	–
	Rape	3631	99.75	0.22	0.03	–	–
Chronicity score*		**N**	**Median**	**Min**	**Max**		
Verbal aggression		3082	26.5	1	87.5		
Physical aggression		2473	11	1	87.5		

*Median number of times the acts in the scale occurred among those who had observed at least one act of aggression

### Characteristics of nursing homes and subtypes of resident-to-resident aggression

Table 3 outlines nursing home characteristics associated with the occurrence of all types of RRA. A higher proportion of staff working in larger nursing homes reported observing one or more acts of physical, material and sexual aggression compared to staff working in smaller nursing homes. A slightly higher proportion of staff working in nursing homes located in urban and suburban reported one or more acts of material aggression than staff in rural areas. A higher proportion of nursing staff working in dementia special care units reported one or more acts of all types of aggression compared to staff in long- and short-term care units.

**Table 3:** Nursing home characteristics and the occurrence of all types of resident-to-resident aggression, n (%)

Characteristics	Verbal	p*	Physical	p*	Material	p*	Sexual	p*
Size								
Small (≤50 beds)	1560 (87.4)	0.236	1223 (67.1)	**0.003**	420 (22.8)	**0.004**	306 (16.6)	**0.001**
Large (> 50 beds)	1522 (88.7)		1250 (71.8)		476 (26.9)		366 (20.8)	
Location								
Urban	1037 (88.3)	0.925	838 (69.8)	0.160	326 (26.8)	**0.049**	211 (17.4)	0.356
Suburban	1383 (87.9)		1125 (70.4)		400 (24.7)		315 (19.5)	
Rural	662 (87.8)		510 (66.6)		170 (22.0)		146 (18.8)	
Unit								
Short-term care	394 (80.7)	**0.001**	278 (55.9)	**0.001**	81 (16.2)	**0.001**	71 (14.2)	**0.001**
Long-term care	1885 (88.3)		1465 (67.4)		470 (21.4)		356 (16.2)	
Dementia special care	676 (93.8)		637 (87.0)		316 (42.3)		221 (29.6)	

*Pearson’s Chi-square test

Table 4 outlines the differences in number of acts of verbal and physical aggression according to facility characteristics. The Kruskal-Wallis test revealed that nursing staff in larger nursing homes reported a higher number of both verbal and physical aggression than nursing staff in smaller nursing homes. Nursing staff working in urban and suburban areas reported a higher number of verbal and physical aggression than nursing staff working in rural areas. Nursing staff working in dementia special care units reported a higher number of verbal and physical aggression than nursing staff working in short- and long-term care units.

**Table 4:** Number of acts of verbal and physical aggression by nursing home characteristics (median)

Characteristics	Verbal			Physical		
	N	Median	*p*-value*	N	Median	*p*-value*
**Size**						
Small (≤50 beds)	1560	25	**0.041**	1223	10	**0.001**
Large (> 50 beds)	1522	26.5		1250	11.5	
**Location**						
Urban	1037	26	**0.011**	838	11.5	**0.006**
Suburban	1383	27.5		1125	11.5	
Rural	662	24		510	9	
**Unit**						
Short-term care	394	16.5	**0.001**	278	5.25	**0.001**
Long-term care	1885	24		1465	8.5	
Dementia special care	676	44.5		637	21	

*Kruskal Wallis test

## Discussion

Our findings indicate that resident-to-resident aggression is a common problem in Norwegian nursing homes, with almost 90% of nursing staff observing at least one incident of RRA during the past year. Verbal and physical aggression were the most commonly reported types but acts of material and sexual aggression were also reported.

It is difficult to compare our rates to previously reported prevalence rates due to the different study methods used. To the best of our knowledge, only the study by Castle [18] used a cross-sectional survey design of staff to explore the extent and nature of RRA in nursing homes, but this study used a reference period of three months and not the past year. Interestingly, the rates in this US study were higher than those in this study, but the rank order of RRA types was the same.

Verbal aggression is often reported as the most prevalent type regardless of study method used [9, 13, 18, 31, 35]. We found that the most prevalent acts reported were residents arguing, yelling, and making nasty remarks, which is similar to that which nursing staff reported in US nursing homes [18] and assisted living facilities [35]. In a nursing home where residents have limited freedom and live in shared and crowded environments, many minor remarks, arguments and incursions in daily life may lead to adverse consequences such as anxiety, depression, dissatisfaction with life, and social loneliness [18, 27]. Such comments and gestures may seem less severe from an outside perspective, but are still perceived as hurtful and distressful for residents [30].

Furthermore, verbal aggression may escalate to physical aggression and residents may be aggressors and victims in the same situations [16, 23]. We found a high occurrence of residents’ pushing, grabbing, or pinching, which is in line with findings from other qualitative and quantitative studies [13, 18]. Physical aggression may lead to minor injuries such as bruises, hematomas, or lacerations, but also to more severe injuries including fractures and dislocations [10]. Moreover, deaths in institutions are often attributed to natural, undetermined, or accidental reasons, when they may in fact may be the direct or indirect consequences of aggressive injuries [54]. Murphy et al. [12] found that “push and fall” incidents were the most common cause of deaths from RRA in Australian nursing homes. This was also found in a study by Caspi et al. [11], where 44% of incidents resulting in death in US long-term care homes had a description of a “push-fall” episode, and in a study by DeBois et al. [15], where “push-type” incidents were commonly described as a cause of fatal injury in the US National Violent Death Reporting System.

We found a higher rate of material aggression than what Harris et al. [55] reported in their survey of family members in US nursing homes, but our rates were significantly lower than those reported by US nursing aides in the study by Castle [18]. Harris et al. [55] used the term “inadvertently taking things” when describing material aggression, where others have used terms such as “taking possessions” [18] or “stealing things” [35]. One could argue that, in the context of nursing homes, residents taking items like snacks, clothes or magazines may not be classified as “theft”. Nevertheless, the invasion of a person’s privacy may create an unpleasant environment, and in the study by Pillemer et al. [30], residents felt harassed and threatened when co-residents wandered into their rooms and touched or took their personal belongings. Furthermore, one may postulate that having regular visits by a relative would prevent material aggression, although Schiamberg et al. [56] found that emotional closeness to family members increased the likelihood of RRA in nursing homes, and the authors deliberated whether the provision of gifts and other amenities substantiated envy and theft by co-residents.

In line with the US study by Castle [18], we found low rates of sexual aggression, but we also found small rates on the most severe acts of sexual aggression: digital penetration and rape. Compared to other vulnerable target populations, such as children and individuals with mental and/or physical impairments, sexual abuse of older people has been the subject of varying attention [57]. In long-term care, the risk of sexual aggression increases as a function of residents’ dependency of care, protection and safety [57, 58], and sexual aggression is found to be associated with a variety of adverse mental, physical, and social outcomes for both victims and aggressors [58, 59]. Sexuality is a basic human need related to quality of life and emotional well-being, but sexuality in later life is often challenged by ageism and stereotypes [60, 61]. Many people with dementia show an interest in physical closeness and sex but may not have the capacity to consent to sexual contact [62]. This makes it challenging for staff to delicately navigate between resident’s rights to sexually express themselves, but also to protect them from mental and physical harm [58]. A systematic review found that staff members’ higher levels of knowledge of older people’s sexuality correlated with positive attitudes towards sexuality in nursing homes [61].

When separating our rates according to facility characteristics, we found more observations of aggression in larger than in smaller institutions, which is in line with the findings by Murphy et al. [12] in Australian nursing homes. Previous studies have found that environmental factors such as a lack of space and crowded areas are triggers of RRA [36], and some larger institutions may have less space per resident compared to smaller nursing homes. We found more observations of aggression in nursing homes located in urban/suburban than in rural located municipalities, which is consistent with the Australian study by Murphy et al. [12] who found more incidents of RRA in metropolitan than in non-metropolitan areas. A possible explanation may be that larger nursing homes are located in urban/suburban municipalities. Nevertheless, these differences in both size and location are not easily explained and should be further explored. Finally, we found more observations of RRA in dementia special care units than in short- and long-term care units, which is consistent with the study by Shinoda-Tagawa et al. [10], who reported that residents in Alzheimer’s disease units were almost three times more likely to be injured by RRA compared to residents in other units. This is not a surprising finding considering that many special care units are specially designed for people diagnosed with Alzheimer’s disease or related dementias that experience severe neuropsychiatric symptoms [42, 63].

Our study has certain strengths and limitations. Firstly, more of the larger nursing homes rejected participation in the recruitment phase, and one could speculate whether these homes were more “problematic” than those who participated. However, they did not differ in how they were run or located. Secondly, several limitations of the study design may have led to an over- or underestimation of the occurrence of RRA. Our findings were based on observations and reports by nursing staff, which may have led to recall bias when remembering incidents in the past year, and a response bias such as a social desirability not to report sensitive acts of aggression. Moreover, staff are not present in all situations in a nursing home, leaving incidents of RRA unwitnessed and unreported [11, 15]. Further contributing to underreporting, one may assume that nursing staff working full-time witness more events of RRA than staff working part-time, and in our study, over half of the participants worked part-time (Table 1). Another bias is that nursing staff working in the same units may have observed and reported the same incidents of RRA. Thirdly, there is no gold standard of survey instruments to measure the prevalence of RRA, and our instrument had only been used in two previous surveys of staff where the psychometric properties had not been evaluated. Consequently, staff may have defined “pushing, grabbing or pinching” as “behaving aggressively toward a resident” and/or “bullying a resident”, increasing the rates of occurrence. Future studies should use factor analysis to evaluate the validity of the survey instrument. Finally, our cross-sectional survey design offers no explanation of causal factors of resident-to-resident aggression in nursing homes.

The strengths of our study are the large sample size of 100 nursing homes and 3693 nursing staff and the high response rate of 60.1%, which allow us to generalise our findings to the rest of the nursing home population in Norway. Moreover, this study is one of the largest staff surveys worldwide to measure the extent and nature of RRA in long-term care facilities.

Detecting the true prevalence of resident-to-resident aggression nursing homes in inherently difficult, and even though our study faces some methodological challenges, we believe the findings provide new knowledge that may have some practical and theoretical implications for care, education, and future research. The CDC states that “*… resident-to-resident aggression … may result when institutions fail to take action to prevent or manage aggression or take actions that are not sufficient to assure resident health and safety”* [14]. Dementia is often highlighted as the ultimate cause of RRA, which undermines the fact that incidents in long-term care settings are often influenced by broader structural conditions and systems [32], which fail to protect and preserve residents in a variety of ways [8, 14, 18]. Several studies have indicated that aggressive behaviours may be the expression of residents’ response related to unmet needs such as hunger, pain, personal care, or sexuality, etc. [17, 26, 36, 62], which could be recognised and managed by use of a more “person-centred” approach that identifies the intrinsic value and uniqueness of each individual [26, 32]. Thus, many healthcare staff recognise behaviours of RRA as normal, acceptable and unchangeable [13], which emphasises the need for knowledge and educational programs that make staff better trained to recognise, manage and report RRA [23, 25, 64]. A promising staff training program (SEARCH approach) by Teresi et al. [24] found a significant increase in knowledge, recognition and longitudinal reporting of RRA by staff in the intervention group compared to staff in the control group. To manage behavioural and psychological symptoms of dementia that often result in episodes of RRA, a study by Lichtwarck et al. [65] found that a multicomponent biopsychosocial approach (TIME) significantly reduced the agitation of residents in nursing homes. Moreover, staff who used TIME experienced increased coping in their approach to residents with complex neuropsychiatric symptoms [66]. In addition to educational programs for staff, nursing homes should emphasise on procedures and structures within the organisation e.g. roommate reassignments, physical space, and removing items that can be used as weapons [13, 15, 36]. Furthermore, some researchers hypothesise that single rooms for nursing home residents may reduce the incidence of RRA [11, 15]. Nevertheless, we need more research on the underlying risk factors within all levels of the social-ecological model, to appropriately design preventive actions.

## Conclusions

We believe our study provides new knowledge concerning the extent and nature of resident-to-resident aggression in nursing homes. Our findings may be important for future international comparability and research, and when designing interventions and strategies to improve the quality of life and safety of nursing home residents.

## Data Availability

The dataset used and analysed during the current study are available from the corresponding author on reasonable request.

## Abbreviations

CDC: Centers for Disease Control and Prevention
RRA: Resident-to-resident aggression
NPS: Neuropsychiatric symptoms
RN: Registered nurse
NH: Nursing home
US: United States
REC: Regional Ethics Committee
SD: Standard deviation
SEARCH: Support, Evaluate, Act, Report Care plan, and Help to avoid
TIME: Targeted Interdisciplinary Model for Evaluation and treatment of neuropsychiatric symptoms
NTNU: Norwegian University of Science and Technology
